# Unusual Left Superior Vena Cava, Connected to the Left Atrium via the Left Superior Pulmonary Vein

**DOI:** 10.34172/aim.2022.111

**Published:** 2022-10-01

**Authors:** Abdelkader Boukhmis, Mohamed El-Amin Nouar

**Affiliations:** ^1^Department of Cardiac Surgery, University Hospital Center MUSTAPHA, Algiers, Algeria

**Keywords:** Cardiopulmonary bypass, Cyanosis, Persistent left superior vena cava

## Abstract

Persistent left superior vena cava (LSVC) that drains into the left atrium (LA) via the left superior pulmonary vein (LSPV) is a rare systemic venous drainage anomaly. It can cause cyanosis and unexplained recurrent strokes. Undiagnosed, it can seriously disrupt the conduct of the cardiopulmonary bypass (CPB), causing sudden air lock and/or flooding of the operative field with venous blood. Its connection with the LSPV outside the pericardium makes its intraoperative diagnosis difficult. We report here the case of a 48-year-old man operated for mitral and aortic valve endocarditis, complicating a Laubry-Pezzi syndrome. The opening of the LA was followed immediately by the entrance of high volume of air bubbles into the superior vena cava cannula which resulted in sudden air lock of the venous outflow line. After multiple lowerings and cessations of pump flow, partial clamping of this cannula resulted in flooding of the LA with venous blood coming from the LSPV. The heart luxation did not allow us to find the LSVC in its usual intrapericardial location, between the LSPV and the left appendage. We had to widely open the left pleura to expose its completely extrapericardial path and its communication with the LSPV. The LSVC was temporally clamped during the remainder of the surgical procedure, then ligated at both ends. The patient underwent mitral valve repair, closure of the infundibular septal defect, aortic valve replacement and tricuspid annuloplasty. He was discharged 10 days later.

## Introduction

 Persistent left superior vena cava (LSVC) that connects with the left atrium (LA) through a left superior pulmonary vein (LSPV) may result in a significant right-to-left shunt. It can cause a multitude of clinical aspects, such as cyanosis or unexplained recurrent strokes.^1^ Undiagnosed; it can seriously disrupt the conduct of cardiopulmonary bypass (CPB). In the present report, we describe a clinical case corresponding to this exceptional variant of the LSVC. This anomaly of the systemic venous drainage was discovered intraoperatively following a sudden air lock. Its connection with the LSPV outside the pericardium made its intraoperative diagnosis and management difficult.

## Case Report

**Figure 1:**
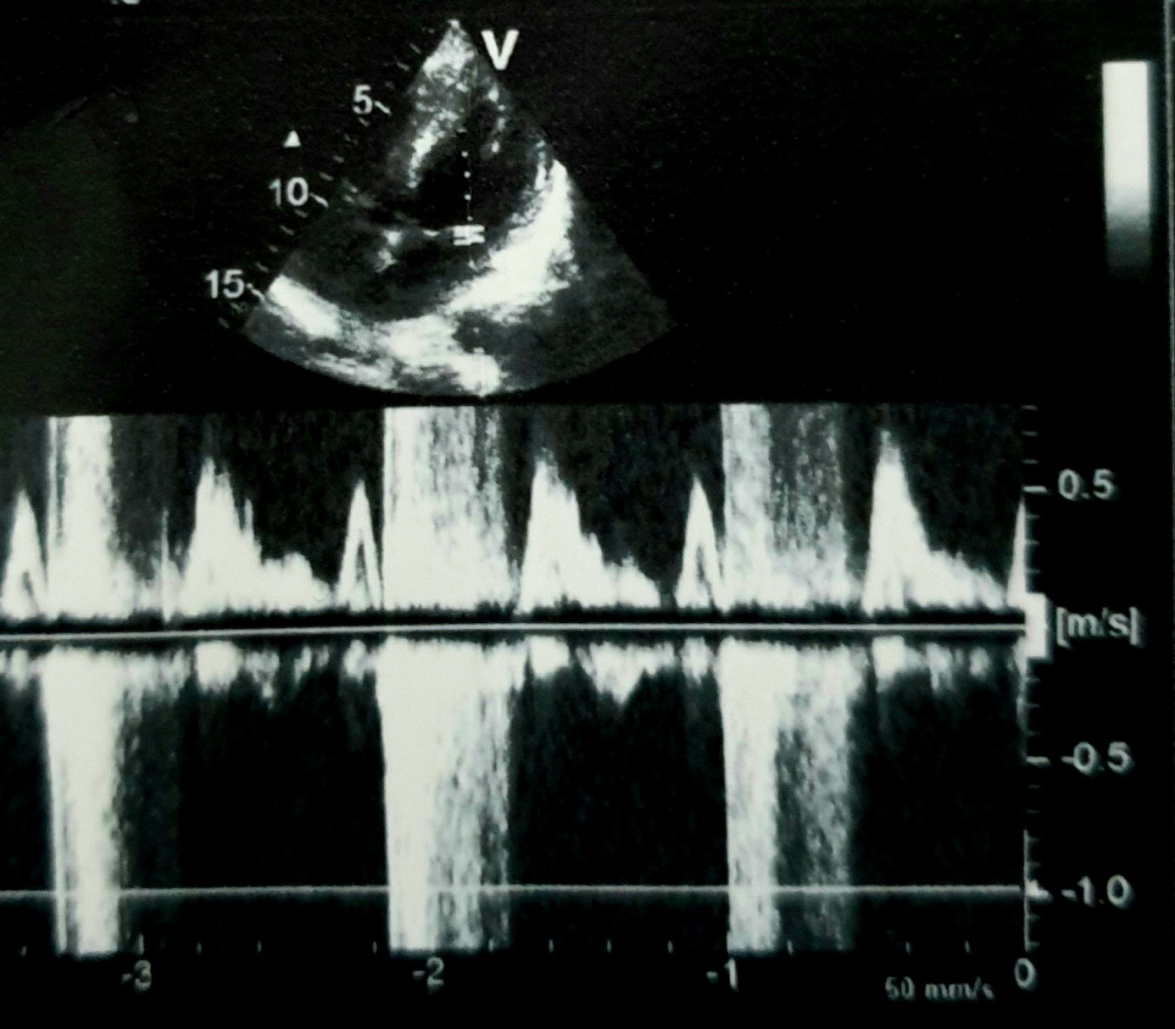
Transthoracic Echocardiography Showing a Ventricular Septal Defect, and Severe Mitral Regurgitation

 A 48-year-old man was referred for surgical treatment of mitral and aortic valve endocarditis, complicating a Laubry-Pezzi syndrome. Transthoracic echocardiography objectified severe aortic regurgitation with mobile vegetations measuring 10 and 20 mm, a left-to-right shunt through an infundibular ventricular septal defect (VSD) (Figure 1), severe mitral regurgitation; poor left ventricular ejection fraction (40%), high pulmonary hypertension (72 mm Hg), and a moderate secondary tricuspid regurgitation. The coronary sinus (CS) was not dilated.

 The patient was operated via a median sternotomy, and CPB was established using aortobicaval cannulation. Cardiac arrest was induced by cold blood cardioplegia, which was delivered through the coronary ostia. The vegetations and damaged aortic valve cusps were excised and closure of the infundibular VSD was performed using an autologous pericardial patch. The opening of the LA and the exposure of the mitral valve were followed immediately by the entrance of high volume of air bubbles into the superior vena cava cannula which resulted in sudden air lock of the venous outflow line. After multiple lowering and cessations of pump flow, partial clamping of this cannula, in order to reduce the inlet air flow, resulted in flooding of the LA with venous blood coming from the LSPV.

**Figure 2:**
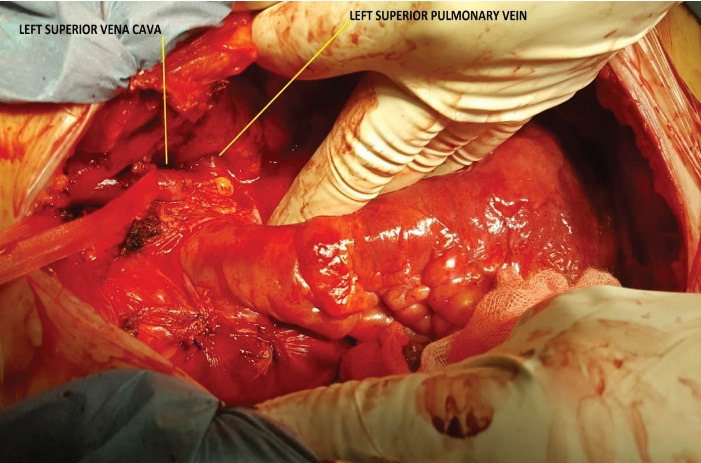
Connection between the left superior vena cava (LSVC) and the left superior pulmonary vein, which drains into the left atrium. Exposure of the totally extrapericardial path of the LSVC after wide opening of the left pleura

**Figure 3:**
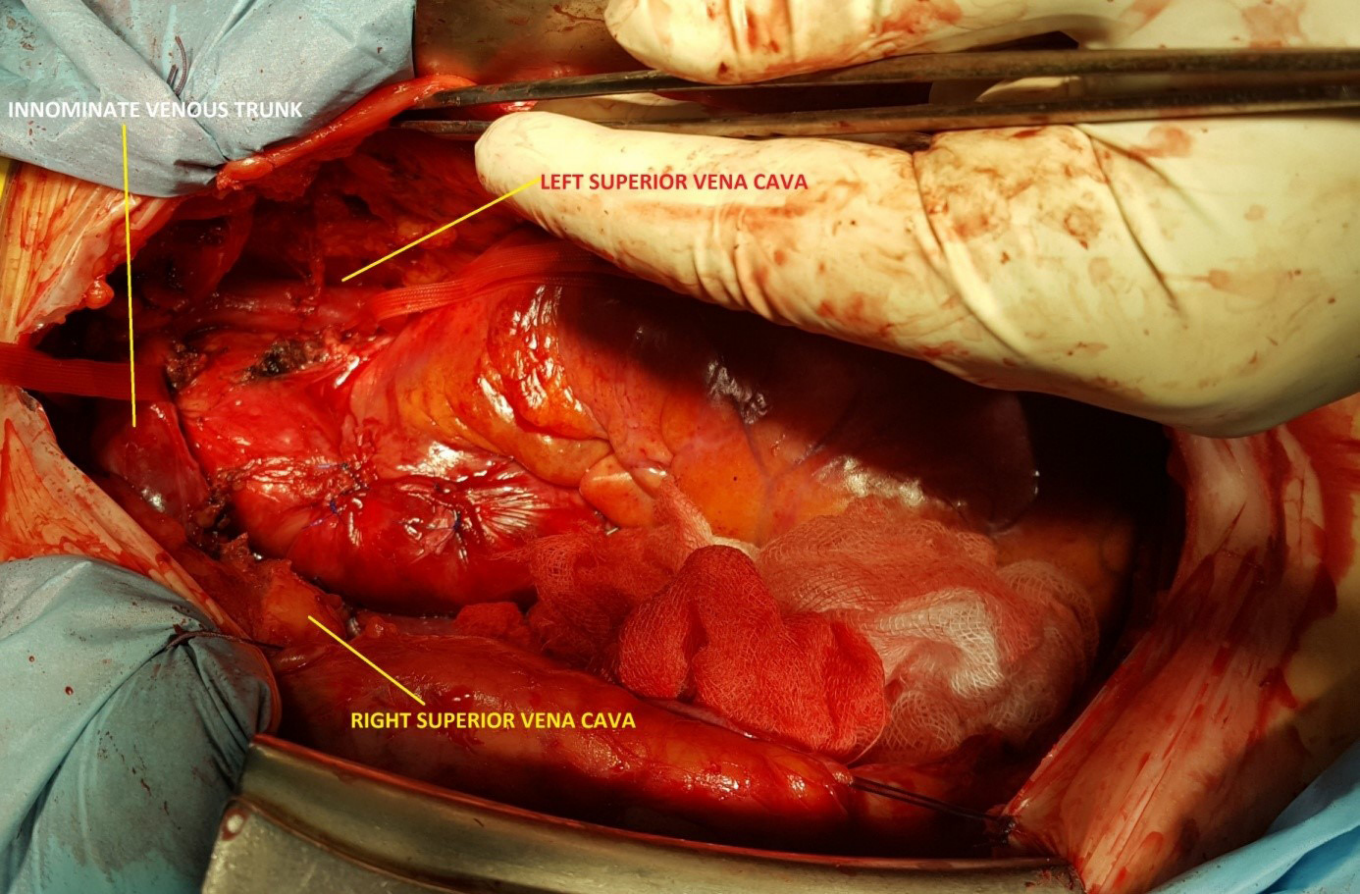
Connection between the left superior vena cava and the innominate vein which is of normal size. Note the presence of a right superior vena cava draining into the right atrium

 Right atriotomy ruled out the existence of an unroofed CS, by verifying the absence of any interatrial communication through the CS. We then temporarily closed the LA before reestablishing full bypass and looking for a persistent LSVC. Since the luxation of the heart did not show a LSVC in its usual place, we widely opened the left pleura for further exploration. This allowed us to discover an exceptional variety of LSVC draining into the LSPV, which pierced the pericardium to drain into the LA (Figure 2). This LSVC communicated at its origin with an innominate vein of normal size, and then descended vertically on the left side of the aortic arch (Figure 3). In order to eliminate any risk of recurrence of air lock of the venous outflow line, we snared the LSVC by a tape mounted on a tourniquet. This allowed full CPB, repair of the mitral valve using Alfieri’s edge to edge technique, and implantation of a mechanical aortic valve prosthesis. We finally performed a De Vega tricuspid annuloplasty on a beating heart. After weaning from CPB, we proceeded to double ligation of the LSVC at both ends. The patient was discharged 10 days later.

## Discussion

 The LSVC is more common than is often assumed. It affects up to 0.3% of the general population and up to 10% of patients with congenital heart disease.^2^ It results from a failure of closure of the left anterior cardinal vein during cardiac development.^3^ In 90% of cases, the LSVC has no hemodynamic consequence. It drains the left jugular and subclavian veins, and then descends vertically on the left of the aortic arch. It pierces the pericardium to penetrate the LA between the pulmonary veins and the atrial appendage, and then continues with the CS which drains into the right atrium.^3^

 In less than 10% of cases, the CS is unroofed and the LSVC drains directly into the LA.^4^ Exceptionally, as is the case with our patient, the LSVC does not pierce the pericardium and drains directly into a LSPV which is normally connected to the LA.^5^

 These last two anatomical varieties lead to a significant right-to-left shunt which can manifest clinically by cyanosis,^6^ recurrent paradoxical thromboembolism,^5^ and air or septic embolism.^7^

 LSVC should be carefully differentiated from partial anomalous pulmonary venous drainage of the left upper lung lobe. In this case, the LSPV is not connected to the LA and drains directly into the persistent LSVC, thus causing a left-to-right shunt.^8^

 The use of contrast transthoracic and transesophageal echocardiography with microbubble contrast agent (agitated saline) application through the left arm intravenous line, can ascertain the presence of this right-to-left shunt. In that case, microbubbles would enter LA by the LSPV, mimicking an intrapulmonary shunt.^9^ Computed tomography or magnetic resonance imaging (MRI) of the chest are recommended if persistent LSVC with atypical left atrial drainage is suspected.

 Endovascular treatment of symptomatic patients with a persistent LSVC and significant right-to-left shunt, using the Amplatzer Occluder^1,10^ or coil embolization^5^ can be an alternative to surgery but are only sporadically reported in the literature.

 When diagnosis of LSVC connected to the LSPV is made preoperatively, the anesthesiologist should not place the central venous catheter on the left internal jugular vein because it can finish its course in one of the branches of the lingular vein. Administration of vasopressors through this catheter could result in pulmonary vasoconstriction and infarction.^11^ Furthermore, transient ischemic attacks or stroke by air embolism can occur after injection of the flush solution into the left arm peripheral intravenous line.^10^

 The cardiologist must rule out the possibility of drainage of the LSVC in the CS, before transvenous pacemaker placement in the left subclavian vein which may cause CS thrombosis in this situation.^12^

 In cardiac surgery, it should be noted that retrograde cardioplegia will be ineffective in cases of LSVC draining in the CS.^13^

 Surgery of the LSVC depends on whether or not it communicates with the right superior vena cava and also on how it enters the heart chambers:

- When the two superior venae cavae communicate via a left brachiocephalic vein (innominate vein) and the LSVC drains into a large CS, which drains normally into the right atrium, it must be preserved if a right atriotomy is not required. Otherwise, it is often necessary to temporarily clamp the LSVC, if the clamped pressure is not higher than 16 mmHg, or to cannulate it through the CS in the opposite case.
- On the other hand, if the LSVC drains into the LA, either directly or through an LSPV, its ligation is necessary before initiating the CPB, thus eliminating the right-to-left shunt without disturbing the cerebral venous drainage.
- If the two venae cavae are not connected, a LSVC draining into the right atrium via an intact CS must be preserved if there is no need to open this chamber. Otherwise, its clamping is prohibited and it must be imperatively cannulated, either through the CS or directly on its extracardiac path. In the case of a LSVC draining into the LA directly (unroofed CS), or indirectly (connection with the LSPV), correction can be achieved by re-routing the left superior vena caval flow into the right atrium using intra-atrial baffle or tunnel techniques.^14^ The anatomy between the orifices of LSVC and pulmonary veins can render these procedures more complex, thus causing a disturbed venous flow.^15^ Recently, extracardiac anastomosis^15^ has been introduced as an alternative technique: (*a*) Anastomosis of the transected LSVC to the tip of the right atrial appendage; (*b*) End-to-side anastomosis of the transected LSVC to the base of the right superior vena cava passing under the aortic arch; (*c*) End-to-side anastomosis of the proximal LSVC to the superior aspect of the left pulmonary artery.

In conclusion, an undiagnosed LSVC connected to the LA, directly in case of an unroofed CS, or indirectly through a LSPV, is usually responsible for flooding the LA and operating field with venous blood, but can also cause sudden air lock, and seriously disrupt the conduct of a CPB.

 LSVC should be sought, not only inside but also outside the pericardium, as is the case of its connection with a LSPV.
